# Direct observation of ionic structure at solid-liquid interfaces: a deep look into the Stern Layer

**DOI:** 10.1038/srep04956

**Published:** 2014-05-22

**Authors:** Igor Siretanu, Daniel Ebeling, Martin P. Andersson, S. L. Svane Stipp, Albert Philipse, Martien Cohen Stuart, Dirk van den Ende, Frieder Mugele

**Affiliations:** 1 Physics of Complex Fluids Group and MESA+ Institute, Faculty of Science and Technology, University of Twente, PO Box 217, 7500 AE Enschede, The Netherlands; 2 Nano-Science Center, Department of Chemistry, University of Copenhagen, Universitetsparken 5, 2100 Copenhagen, Denmark; 3 Van't Hoff Laboratory for Physical and Colloid Chemistry, Debye Institute, Utrecht University, Padualaan 8, 3584 CH Utrecht, The Netherlands; 4 These authors contributed equally to this work.

## Abstract

The distribution of ions and charge at solid-water interfaces plays an essential role in a wide range of processes in biology, geology and technology. While theoretical models of the solid-electrolyte interface date back to the early 20^th^ century, a detailed picture of the structure of the electric double layer has remained elusive, largely because of experimental techniques have not allowed direct observation of the behaviour of ions, i.e. with subnanometer resolution. We have made use of recent advances in high-resolution Atomic Force Microscopy to reveal, with atomic level precision, the ordered adsorption of the mono- and divalent ions that are common in natural environments to heterogeneous gibbsite/silica surfaces in contact with aqueous electrolytes. Complemented by density functional theory, our experiments produce a detailed picture of the formation of surface phases by templated adsorption of cations, anions and water, stabilized by hydrogen bonding.

Gouy^1^, Chapman^2^ and Stern^3^ laid the foundation for our understanding of the electric double layer by describing the distribution of ions in the vicinity of charged interfaces using Poisson-Boltzmann theory. The classical approach has been refined in many respects, including a variety of sometimes competing microscopic effects, such as preferential binding to specific surface sites^4^,^5^, dispersive ion-substrate interactions^6^ and ion correlation effects^7^. More recently, molecular simulations have contributed additional insight, e.g. about the hydration of ions and surfaces. In comparison, common experimental methods such as batch titrations, electrokinetic and surface force measurements provide less direct information on the atomic scale. They integrate laterally over rather large and frequently very heterogeneous surface areas and rely on a large number of assumptions and empirical parameters to fit to theoretical models. Also, along the direction normal to the surface, these techniques average information and attribute it to several of the levels in the electric double layer, based on conceptual model assumptions. It is increasingly recognized^8^,^9^ that quantitative understanding of mineral-fluid interface behaviour is limited because experimental techniques have not been able to capture the complex structure of solid-liquid interfaces with resolution at nanometre scale, parallel and perpendicular to surfaces.

Atomic Force Microscopy (AFM) has recently been advanced to a stage that allows for imaging solid-liquid interfaces at ‘true’ atomic resolution^10^,^11^,^12^,^13^. We have used small amplitude dynamic AFM to explore the surfaces of synthetic nanoparticles of gibbsite (α-Al(OH)_3_)^14^ during exposure to a variety of electrolyte solutions. We chose gibbsite because it can be synthesised reproducibly, to yield suspensions of essentially monodispersed particles. Moreover gibbsite is a good model for some clay mineral surfaces^15^. Sorption of inorganic and organic ions to Al (hydr)oxides, such as gibbsite, and to clay minerals is important for the transport of contaminants and nutrients in the environment and kaolinite, a clay with one Al-OH surface has been reported to play a role in enhancing oil recovery^16^,^17^. It has long been assumed that the doubly coordinated Al_2_OH groups on gibbsite basal planes are inactive to deprotonation/protonation reactions and that surface charge and ion sorption are dominated by the singly coordinated aluminol at edges^18^. Recently however, experimental^8^,^19^,^20^,^21^ and numerical studies^21^,^22^ have suggested that missing information about structure at the submicrometre scale and the ratio of edge to basal surface area might have compromised data interpretation.

With small amplitude dynamic AFM, we have collected the required high resolution insight needed for addressing these questions, to directly “see” the structure of the ions adsorbed in the Stern layer and to observe changes in the pattern on the gibbsite basal plane as the contacting solution is changed. We characterised the surfaces at two levels. First, we used AFM spectroscopy at tip-sample distances of a few nanometres during exposure to solutions with a range of concentrations. This provides data on effective surface charge, similar to those obtained by *ζ*-potential measurements. Next, we recorded atomic scale images at much smaller distances, which gives a direct view of ion distribution within the Stern layer. Finally, using density functional theory, we could confirm the stability of the ordering observed by AFM and gain additional insight into the nature of the bonding and how charge in the Stern layer changes with solution composition.

## Results

**Figure 1:**
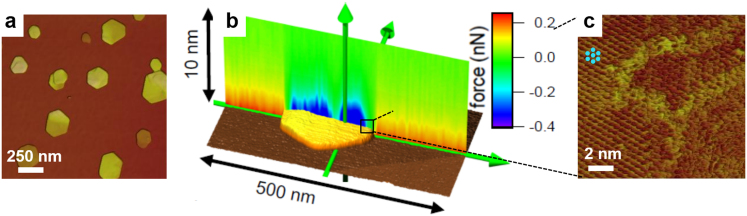
Atomic force microscopy (AFM) of gibbsite nanoparticles. (a), Topography images of gibbsite on an oxidised silicon wafer. (b), color-coded 2D force field generated from 100 tip-sample interaction curves in 20 mM NaCl at pH ≈ 6. (blue: attractive force; red: repulsive force; green: zero force; see scale bar) (c), Amplitude modulation atomic resolution image of a gibbsite particle in ultrapure deionised water. Left part: pseudohexagonal basal plane structure (surface unit cell, *a* = 0.87 nm, *b* = 0.50 nm); centre: atomic step disorder on terrace edges; bottom right: edge of the particle.

The gibbsite particles were deposited from a water-ethanol solution (details in Methods Section) on silica wafers that had oxidised in air to produce amorphous SiO_2_. The nanoparticles naturally sorb with their {001} basal plane adjacent to the silica surface, exposing a Al-OH surface to the solution. Typically, gibbsite particles attached singly. Lateral dimensions ranged from several 10 to a few 100 nanometres and heights, from 1 to 20 nm (Fig. 1a). All experiments were performed in slightly acidic (pH ~ 6) aqueous electrolyte and we used silicon AFM tips. Tip surfaces had oxidised to amorphous silica so they had the same character as the silica substrate.

We monitored the effective surface charge of the particles by measuring the interaction force between the tip and sample as a function of distance, in frequency modulation force spectroscopy mode^23^ (FM-AFM). Two dimensional interaction force maps^24^ (colour coded in Fig. 1b) confirm that on the silica substrate, force increases from zero (green) to repulsive values (red) of several hundred picoNewtons at a distance of several nanometers, as expected for two negatively charged surfaces in pure water. Over the gibbsite particles however, attractive force (blue) indicates positive charge. Compared with the silica substrate, there is more lateral variation in the force on the gibbsite particles, indicating a larger degree of heterogeneity. Force decreases toward the particle edges. Force profiles (Fig. 1b) also reveal the location of occasional crystal defects. The local minimum in the attractive force near the center of this specific particle is caused by a twin boundary. This is most easily seen in 2D frequency shift images (Fig. S1b) that show a direct, qualitative measurement of the interaction forces. The minimum force indicates that the effective local surface charge essentially vanishes close to the crystal defect. Typical tip radii of 20–30 nm in the spectroscopy experiments imply a lateral average of a few thousand surface unit cells.

**Figure 2:**
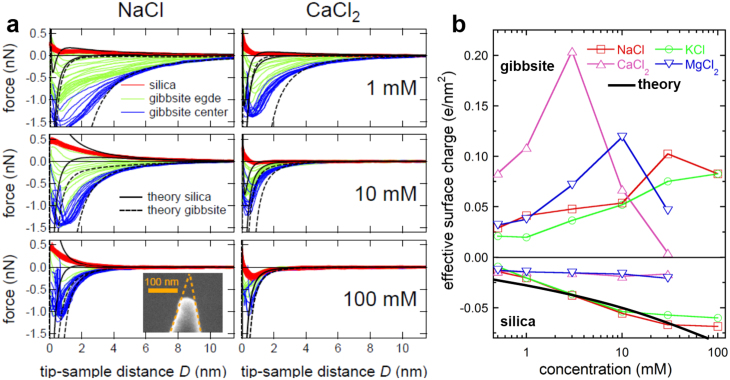
Electrical properties of amorphous SiO_2_ and gibbsite measured with FM-AFM. (a), Force vs distance curves measured over a gibbsite nanoparticle sorbed on oxidised silicon wafers in 1, 10 and 100 mM NaCl and CaCl_2_ solutions. Red curves: tip on silica substrate. Green: edge of gibbsite particle. Blue: centre of gibbsite particle. Lines (solid: silica; dashed gibbsite): tip sample interaction force according to DLVO theory for constant charge (top) and constant potential (bottom) boundary conditions. Inset: SEM image of AFM tip after the experiment. (CFM Aspire tip, with parameters of silicon cantilever f_0_ = 22.9 kHz, c_z_ = 5.0 N/m, Q = 9). (b), Surface charge as function of solution composition (pH ≈ 6). Symbols: experimental data. Solid black line: best fit assuming deprotonation of silanol groups in monovalent salt solutions (see text for details).

Atomically resolved amplitude modulation images of the basal planes display the periodicity of the gibbsite lattice (Fig. 1c). Close to particle edges, we typically observe a higher density of atomic steps. Frequently, these steps are decorated by adsorbed material (Fig. 1c). Such defects are an important source of charge heterogeneity on gibbsite surfaces. Figure 2a shows line representations of force spectroscopy data for areas such as Figure 1b under several concentrations of NaCl and CaCl_2_. Each data set was obtained with the same cantilever and sample and care was taken to guarantee that tip shape did not change when solutions were changed (see Supplementary Information). On silica, the force curves (red in Fig. 2a) from a number of sites collapse into a single narrow band for each ion concentration. The interaction curves for gibbsite are more widely spread, with rather weak forces along particle edges (green) and strong attraction in the centre (blue). Our next discussion focusses on the forces in the centre.

The qualitative trends in Figure 2a follow those expected from standard electrostatic screening, i.e. force decreases as salt concentration increases and the absolute force for divalent ions at the same concentration is lower than for monovalent ions. To determine surface charge, we compare the force curves to predictions from DLVO (Derjaguin-Landau-Verwey-Overbeek) theory^23^ for electrostatic and van der Waals forces (Supplementary Information). Consistent with expectations, forces measured at small separations lie between the two limiting cases of constant charge and constant potential because of confinement induced charge regulation^25^. However, from the asymptotic regime at large separation, we can readily extract unique values for the effective surface charge, *σ*_eff_, for both the tip and sample^24^. For the monovalent salts, *σ*_eff_ on silica increases with increasing salt concentration, whereas for the divalent salts, it remains constant within experimental error (Fig. 2b). This trend for monovalent salts agrees with the expected enhanced deprotonation of silanol groups on the silica surface: ≡ SiOH → SiO^−^ + H*^+^* as electrostatic screening increases. Fitting the data with a basic Stern model (BSM)^25^ yields pKa ~7.5 for silanol deprotonation, in good agreement with literature data^25^,^26^,^27^,^28^,^29^ (black line in Fig. 2b). This supports the effectiveness of our measurement and data analysis procedure. Weakly negative and essentially constant surface charge on the amorphous silica surface in contact with Ca^2+^ and Mg^2+^ has previously been interpreted in terms of cation adsorption^27^,^30^.

On gibbsite, *σ*_eff_ was positive under all investigated conditions. In solutions of monovalent salts, it increases monotonically from ~0.03 to ~0.1 e/nm^2^ as salinity increases. The surface unit cell has an area of ~0.44 nm^2^ so these absolute values imply that at most, a few percent of the unit cells carry a net charge. A more intriguing behaviour is observed for the divalent cations. Initially, *σ*_eff_ increases strongly with increasing salinity, reaching a maximum at 5 to 10 mM and then decreases to negligible values as concentration reaches 100 mM. A slight but consistent specific ion effect was observed in three separate experiments. In CaCl_2_ solutions, maximum charge is higher and it occurs at somewhat lower concentration than in MgCl_2_. While the constant increase in *σ*_eff_ for monovalent salts could be interpreted to result from protonation facilitated by improved screening, as we see for silica^9^,^31^,^32^, the behaviour of divalent cations is more complex. (Fitting the data for the monovalent salts in terms of a simple surface speciation model involving protonation of doubly coordinated Al_2_OH groups at low pH yields a pKa value of ~7 and a density of one active group per surface unit cell, reasonably consistent with recent models of the gibbsite surface). The increase and decrease suggests the presence of two separate processes. The first process, dominant at lower salt concentrations, enhances the already positive effect of surface charge. The second reduces it again. Obviously, the first process cannot be driven by electrostatic forces, the second one might be.

**Figure 3:**
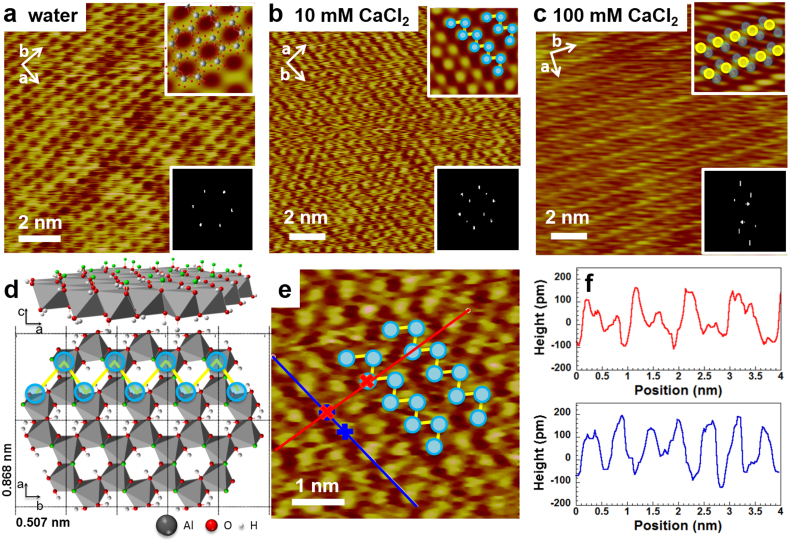
Atomic resolution AFM images of gibbsite. (a), AFM topographic image of gibbsite basal plane in ultrapure deionised water. Insets: zoomed and Fourier-filtered view with superimposed crystallographic lattice (top); 2D fast Fourier transform of image. (b), same type of data recorded in 10 mM and (c), 100 mM CaCl_2_ solution. (d), Crystal structure of gibbsite in *ac* and *ab* planes. H atoms pointing perpendicular to the *ab* plane are shown in green. (e), A zoom view of b with schematic indication of position of adsorbates and location of the height profiles in *a* (red) and *b* (blue) directions shown in f. Height profiles in *a* (red) and *b* (blue) directions display periodicities of 0.87 nm and 0.50 nm, respectively.

At this stage, it is tempting to invoke possible adsorption/desorption reactions to explain Figure 2b. The rather low absolute value of *σ*_eff_ is consistent with general understanding, that the Al-OH gibbsite basal plane is indeed chemically rather inactive^18^,^19^,^20^. However, atomic force spectroscopy, just as electrokinetic measurements, probes the charge in the diffuse part of the electric double layer. These techniques might be too indirect to deliver a detailed picture of the complex chemical processes that take place at the solid-liquid interface. To overcome this limitation, we imaged the gibbsite surface at atomic resolution under several electrolytes (details in Methods). Figure 3a shows the typical pseudohexagonal pattern of the gibbsite basal plane, imaged under deionized water. The pattern is caused by the arrangement of the octahedral cavities with next neighbour spacing of ~0.5 nm, consistent with dimensions of the surface unit cell with dimensions *a* = 0.868 nm and *b* = 0.507 nm (Fig. 3d), as obtained by x-ray diffraction. Except for an occasional contrast inversion (Fig. S3), which we attribute to loss of true atomic resolution^12^, symmetry, contrast and the resolution of the pattern remain unchanged when the water is replaced by solutions of KCl or NaCl. From the absence of changes in surface topography, we conclude that neither the monovalent cations nor Cl^−^ adsorbs strongly to the gibbsite surface. Ions could be weakly adsorbed and pushed away by the AFM tip, as has been discussed for mica in contact with electrolyte solutions^12^,^33^,^34^,^35^. Nonadsorption of monovalent ions is completely consistent with protonation as an explanation for the increase in effective surface charge, discussed above.

In stark contrast to behaviour in monovalent salt solutions, gibbsite appearance changes dramatically when the solution is replaced with 10 mM CaCl_2_ or MgCl_2_ (Fig. 3b and Fig. S4b). The pseudohexagonal pattern gives way to an array of double rows aligned along the *b* direction (Fig. 3b and e). Each double row consists of alternating bumps. The periodicity along and perpendicular to the double rows is 0.50 nm and 0.87 nm (Fig. 3f), in excellent agreement with the surface atomic structure. There are thus two bumps per surface unit cell, which we interpret to be (possibly hydrated) ions adsorbed from solution.

**Figure 4:**
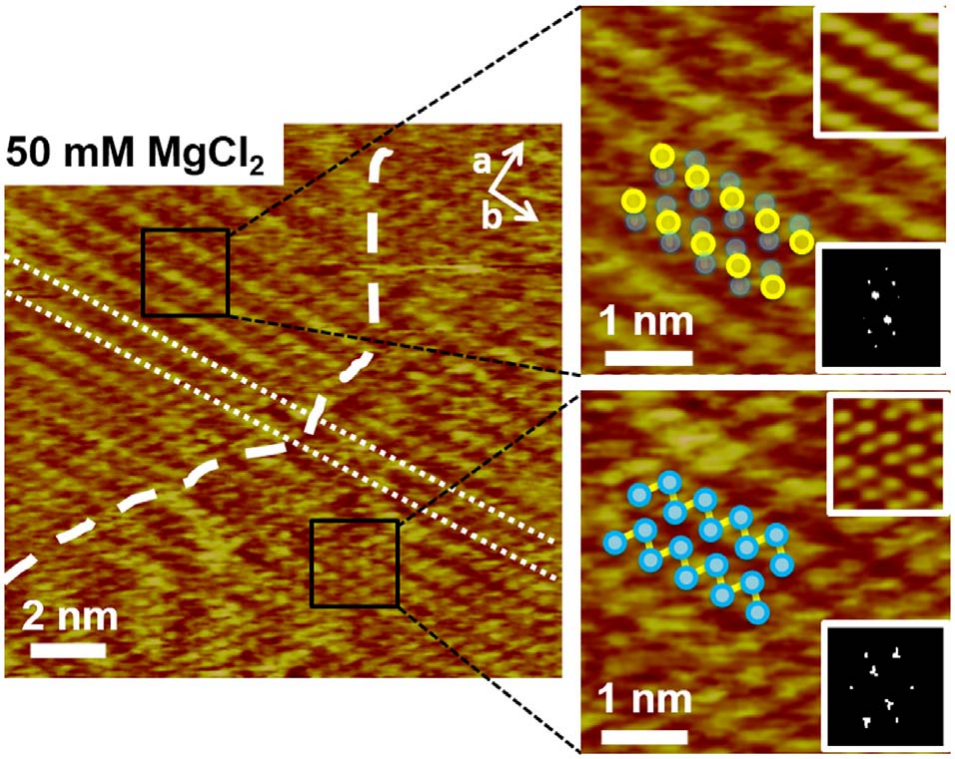
Gibbsite imaged in 50 mM MgCl_2_ showing phase-separated domains with double row structure (bottom right) and single row structure (top left) characteristic of low and high salt concentrations. The area to the right of the white dashed line has equivalent height double rows in a zig zag pattern with the same periodicity as Figure 3e and all images obtained under 10 mM CaCl_2_ or MgCl_2_ solutions. Left of the dashed line, the rows alternate in height, as observed for all of the surfaces imaged under solutions of 100 mM CaCl_2_ or MgCl_2_.

As we increase the concentration of CaCl_2_ to 100 mM, we observe a second change in the appearance of the surface. The double rows give way to single rows spaced by one lattice vector along the *b* direction and with one bump per surface unit cell along the *a* direction (Fig. 3c). In between two adjacent rows, a second row of bumps is sometimes seen, typically at much fainter contrast. The same behaviour is observed when gibbsite is exposed to MgCl_2_ solutions (Fig. S4c). At intermediate concentrations (≈50 mM), we see coexisting domains of double rows and of alternating bright-faint rows (Fig. 4). This suggests two distinct two dimensional adsorbed phases.

At this stage, we can already conclude that the gibbsite basal plane is by no means chemically inactive. Rather than occasional reaction of a few percent of the surface unit cells, as suggested by the low value of *σ*_eff_ and generally assumed in the literature^5^,^18^,^19^,^20^, our images show that every unit cell accepts at least two adsorbed ions, where the bond is strong enough that it is not pushed away by the tip. The concurrent appearance of the (positive) maximum in *σ*_eff_ and the double rows in the high resolution images suggests that both phenomena result from adsorption of the same type of ion. Because Cl^−^ does not affect the surface pattern, even at concentrations of 100 mM NaCl or KCl, we conclude that the double rows must be caused by divalent cation adsorption. The agreement of the measured periodicities of the double row structure with the surface unit cell dimensions suggests bonding to well defined adsorption sites, rather than electrostatic correlation between ions^36^. To identify the adsorption sites, we can look more closely at the surface structure. The gibbsite surface unit cell has six chemically inequivalent Al_2_-OH moieties. Simulations suggest that deprotonation of these sites covers a rather wide range of pK_a_^37^. Three of them are located around the central octahedral cavity and point toward the solution (small green dots in Fig. 3d). These OH groups are available for interlayer hydrogen bonding in the bulk gibbsite structure^38^ and for hydrogen bonding to adsorbates at the surface^39^,^40^,^41^. Attachment at these sites would produce the observed dimensions and zig zag pattern (Fig. 3d).

The simultaneous decrease of surface charge and change in pattern appearance at higher concentrations suggest adsorption of Cl^−^ ions. While there is no evidence for Cl^−^ adsorption on gibbsite, chloride interaction with adsorbed Ca^2+^ and Mg^2+^ could promote attachment. As concentrations increase, both Ca^2+^ and Mg^2+^ form ion pairs with Cl^−^ so pairing on surfaces is consistent. Chloride adsorption has recently been reported in molecular dynamics simulations of smectite-electrolyte interfaces^42^.

The adsorption of two divalent cations per unit cell without any compensation of charge through surface deprotonation or coadsorption of anions corresponds to a hypothetical surface charge of 9.2 e/nm^2^. This is inconsistent with the low values of *σ*_eff_ (Fig. 2b), which correspond to less than one elementary charge per unit cell. Substantial deviations between surface charge determined by macroscopic methods such as titration and values obtained from the diffuse layer, for example by electrokinetic or force measurements, are not uncommon^43^. They are generally attributed to uncertainties in the exact location of the shear plane in electrokinetic measurements and the mobility of weakly adsorbed ions. The mismatch in charge density could originate from surface deprotonation or adsorbed anions, that contribute to the effective surface charge in spectroscopy experiments but that are too mobile to remain localized under high-resolution imaging.

**Figure 5:**
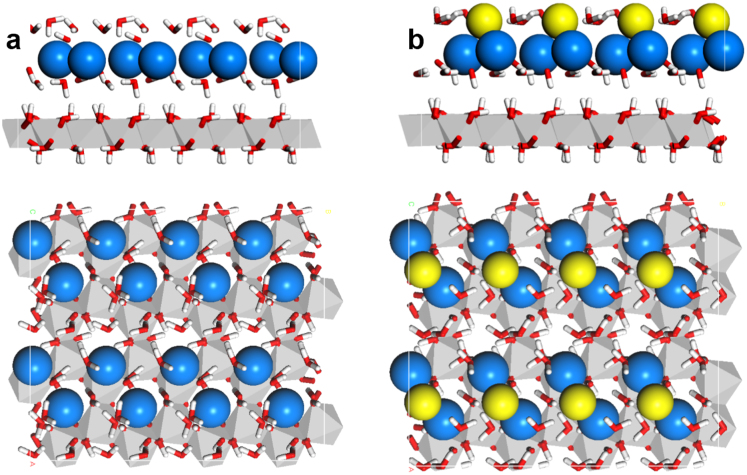
Equilibrium structure of adsorbed Ca^2+^ (blue) and Cl^−^ (yellow) on the gibbsite basal plane in contact with aqueous solution predicted by DFT calculations. Red and white: oxygen and hydrogen; gray: Al-O octahedral. (a), Side and top view of the optimized geometry for outer shell adsorption of Ca^2+^ (blue) on gibbsite. A 2 × 2 supercell of our simulation cell is shown for clarity. (b), At higher concentrations of CaCl_2_. Adsorption plane of Cl^−^ is 0.22 nm above Ca^2+^.

For a more detailed analysis of bonding tendencies and to help explain the surface charge behaviour, we used density functional theory (DFT) to examine the adsorption of Ca^2+^, Mg^2+^ and Cl^−^ onto the Al-OH basal plane of gibbsite. We use the COSMO-RS implicit solvent model with periodic boundary conditions to calculate the equilibrium structure of the adsorbed divalent cations for both outer and inner sphere configurations, i.e. with or without water of hydration between the ion and the surface (details in Methods and Supplementary Information). In both cases, stable zig-zag double rows were found. However, only formation of an outer sphere configuration, containing enough hydration water to retain the average bulk ion-water coordination number of six, was exothermic. The formation energies for the divalent ion structures were −118 kJ/mol/Ca(OH)_2_ and −115 kJ/mol/Mg(OH)_2_ (Table S1), Figures 5a and S5 show the equilibrium, outer shell configurations that excellently reproduce the experimentally observed double row structure, with alternating adsorption sites. Three of four hydroxyl groups, added to guarantee charge neutrality, act as hydrogen bonding acceptors for surface protons. The fourth OH^−^ bridges between the two cations. It is interesting that the fourth hydroxyl causes a slight asymmetry in the zig-zag, which is compatible with the experimental data (Supplementary Information, Fig. S6a, where structure from Fig. 5a is superimposed on the AFM image).

Although the surface unit cell is charge neutral, our model offers an interesting explanation of the slight positive surface charge at intermediate salinities. The alternating structure of hydrated divalent cations offers several sites where hydration water and OH^−^ bridge between two cations. Water adsorbed on similar sites on calcite surfaces is significantly more acidic than bulk water, with pK_a_ as low as 3 to 4^44^. Additional COMSO-RS DFT calculations for clusters of about 200 atoms, beginning with the converged solution of the periodic calculation, allowed us to calculate pK_a_ of 10.2 and 4.9 for H_2_O → OH^−^ deprotonation for the adsorbed Ca^2+^ and Mg^2+^ structure. These values suggest that the positive charge in the spectroscopy measurements results from partial protonation of hydroxyl that bridges adjacent cations from solution. The pK_a_ for Mg^2+^ adsorption is lower than for Ca^2+^, implying that OH^−^, and hence the electroneutral configuration, is somewhat more stable for Mg^2+^, in agreement with the experiments, which show that the maximum charge for the Mg^2+^ structure is always lower than for the Ca structure, Figure 2b and Figure S2.

Finally, we calculated the equilibrium configurations of the adsorbed cations where one Cl^−^ ion per unit cell replaced the bridging hydroxyl ion. Chloride also bridges adjacent cations, slightly shifted towards the pseudo threefold cavity (Fig. 5b). The vertical position is 210 pm above the plane, averaged over the metal cations (cf. Fig. S6b). This ion exchange disables OH^−^ protonation and results in a neutral surface structure, which explains the decrease in *σ*_eff_ at high salinity. The OH^−^ vs. Cl^−^ exchange energies are +39 kJ/mol and +47 kJ/mol for the Ca^2+^ and Mg^2+^ structures (Table S2). For pH = 6, this implies characteristic concentrations of 30 mM for CaCl_2_ and 900 mM for MgCl_2_ to induce the exchange reaction. These values are in very good agreement with the experimental data and even explain the slight shift of maximum *σ*_eff_ toward higher Mg^2+^ concentrations, compared with Ca^2+^, in Figure 2b.

In conclusion, the combination of AFM spectroscopy, high resolution imaging and numerical simulations provides unprecedented insight into the complex processes involved in the formation of the electric double layer on mineral surfaces. By resolving the internal structure of the Stern layer we demonstrate a strong affinity for divalent cations of a type of surface that has long been assumed to be chemically inactive. For the specific case of gibbsite, the resulting changes in surface chemistry have important consequences for the role of Al-OH bearing mineral surfaces in modern technologies for enhanced oil recovery.

## Methods

### Sample preparation

Gibbsite synthesis is described in detail by Wierenga et al.^14^ We diluted the gibbsite stock suspension 100 times in a 1:1 mixture of ultrapure deionised water (Milli-Q) and ethanol and we deposited 10 ml on freshly cleaned silica substrates. After 30 s, they were rinsed with copious amounts of deionised water and blown dry with air.

### Atomic Force Microscopy (AFM)

For atomic resolution, we used a Multimode8 AFM (Bruker Nano) equipped with Nanoscope V controller and an A scanner, operated in tapping mode, mostly with Bruker FASTSCAN-B cantilevers (f_0_ = 170 kHz, c_z_ = 3 N/m, Q = 10). Controls with Olympus AC-55, Aspire CFM (f_0_ = 22.9 kHz, c_z_ = 5.0 N/m, Q = 9) and CT130 probes yielded similar results. Before use, tips were cleaned by rinsing with a mixture of ethanol and isopropanol (≈1:1) and air plasma treatment (Harrick Plasma) for 15–30 min. A standard tapping mode liquid probe holder without O-ring (Bruker Nano) was used for imaging. To minimize drift, the system electronics were allowed to equilibrate for 20–60 min before data were acquired. The AFM was operated in amplitude-modulation mode with free amplitude, A_0_, typically less than 2 nm, high scan rate, ≈10 Hz, and imaging amplitude set point as high as possible, typically A/A_0_ ≥ 0.8. All images were flattened using Bruker's standard Nanoscope Analysis 1.4 package, including, in some cases slight low pass filtering to improve clarity.

AFM spectroscopy measurements were performed with a Dimension Icon AFM (Bruker Nano) equipped with Nanoscope V controller which does not use a liquid cell exciting “the whole chip”, but rather a direct excitation of the cantilever as in usual dynamic AFM in ambient air^45^. Additional drive electronics (QFM-Module, NanoAnalytics GmbH, Germany) was used to operate the system in constant excitation (CE)^46^ version of the frequency modulation technique and measure the frequency shift of the oscillating cantilever, since it is known to be more robust, especially for liquid applications, because it does not require an additional feedback loop which keeps the oscillation amplitude constant (as in the constant amplitude mode). Spectroscopy measurements were performed with rectangular silicon cantilevers with conical tips (CFM and CT130, Aspire), using the standard direct drive liquid probe holder and 60 mm glass petri dishes for the samples. The petri dishes, tips and silica sample substrates were rinsed with isopropanol, ethanol and MilliQ water before cleaning with air plasma for 15–30 min. To minimize changes in the tip apex during the spectroscopy measurements, we did not allow the amplitude signal to drop below ~70% of its value far away from the surface (free amplitude ≈ 2 nm) by setting a threshold. Tip-sample forces are calculated from the amplitude and frequency shift vs distance curves as described in elsewhere^47^. The measured interaction forces between tip and sample surface are converted to surface charge using Poisson-Boltzmann theory, taking into account the actual tip geometry^23^ (Supplementary Information).

### Computational details

Periodic density functional theory (DFT) calculations were performed using the program DMol3 with the COSMO-RS implicit solvent^48^, the PBE functional, the DNP basis set and a dispersion correction^49^. We used a 1 × 2 gibbsite basal plane unit cell with lattice parameters (0.86840 × 1.01560 nm) defined by x-ray diffraction. Periodic slab calculation included three molecular layers, of which the lowest was frozen during all optimisations. Calculations for predicting pK_a_ for water binding to the adsorbed cations were performed with a cluster of gibbsite {001} containing 204 atoms. The cluster was terminated with hydrogen at the Al-OH edges to ensure electroneutrality for the structure with adsorbed OH^−^. Further description of the experimental and theoretical details is provided in Supplementary Information.

## Author Contributions

I.S. and D.E. performed the experiments; M.P.A. performed the DFT calculations; A.P. synthesised the gibbsite and developed the sample preparation procedures; M.C.S., S.L.S.S., M.P.A., I.S., D.E. and F.M. contributed to discussion and writing; I.S., D.E., M.P.A. and F.M. designed the research.

## Supplementary Material

Supplementary Information Direct observation of ionic structure at solid -liquid interfaces: A deep look into the Stern Layer
