# Supplementary material for: Direct observation of ionic structure at solid-liquid interfaces: a deep look into the Stern Layer
Source: Sci Rep. 2014 May 22;4:4956. doi: 10.1038/srep04956 (PMC4030399; doi:10.1038/srep04956)
Supplement: Supplementary Information — Direct observation of ionic structure at solid -liquid interfaces: A deep look into the Stern Layer [file srep04956-s1.pdf]

# Supplementary Information

# Direct observation of ionic structure at solid - liquid interfaces: A deep look into the Stern Layer

*Igor Siretanu[1†], Daniel Ebeling[1†], Martin P. Andersson[2], S. L. Svane Stipp[2], Albert Philipse[3], Martien Cohen Stuart[1], Dirk van den Ende[1] and Frieder Mugele[1*]*

[1]Physics of Complex Fluids Group and MESA+ Institute, Faculty of Science and Technology, University of Twente, PO Box 217, 7500 AE Enschede, The Netherlands

[2]Nano-Science Center, Department of Chemistry, University of Copenhagen, Universitetsparken 5, 2100 Copenhagen, Denmark

[3]Van't Hoff Laboratory for Physical and Colloid Chemistry, Debye Institute, Utrecht University, Padualaan 8, 3584 CH Utrecht, The Netherlands

## I. Experimental and theoretical details

**Gibbsite synthesis**[1]**:** An aqueous solution of aluminium-iso-propoxide (15.2 g/L), aluminiumsec-butoxide (20 g/L) and HCl (0.86 M) was stirred in a closed vessel at room temperature for 10 days. The resulting turbid solution of amorphous aluminium hydroxide suspension was transferred to an autoclave for 72 h at 85 °C and then put into cellophane dialysis tubes and dialyzed against deionized water for 1 week. The procedure yielded a platelet concentration of 5-8 g/L in the final suspension. The BET ($N_2$) specific surface for the material was 76 $m^2$/g and it did not change during storage of the stock material. Transmission electron micrographs show that colloids are well defined and fairly monodisperse hexagons with an average diameter of 160 nm. Potentiometric titration and the electrophoretic mobility suggest that edges of gibbsite colloids have an isoelectric point close to pH 7, which differs from that on the faces, i.e. pH~10. To prepare the samples for spectroscopy and AFM measurements, the stock suspension was diluted 100 times in a mixture of ultrapure deionised water (MilliQ) and ethanol (1:1). 10 ml of the suspension are deposited onto freshly cleaned silica substrates. After 30 s residence time, the samples is rinsed with copious amounts of ultrapure water and blown dry with air.

**Force spectroscopy:** Measurements were made in a range of solutions made with NaCl, KCl, $MgCl_2 \cdot 6H_2O$, $CaCl_2 \cdot 2H_2O$ purchased from Sigma Aldrich, all at puriss, p.a. or ACS reagent grade. We made a stock solution of 1 M for each salt and diluted it for each experiment to concentrations of 0.5, 1, 3, 10, 30, and 100 mM. For each of the 24 cases, 100 interaction curves were measured along a scan line over a single gibbsite platelet. For each salt, a new, freshly cleaned silica substrate and petri dish were used and the series of measurements began with the sample exposed to the 0.5 mM solution. After completing the spectroscopy measurements for the lowest concentration, the cantilever was retracted from the surface by 0.5 mm, without removing

it from the liquid. The concentration was increased to 1, then 3, 10, 50 and 100 mM in turn, by adding aliquots of the stock solution. At each concentration, the liquid in the petri dish was gently mixed by flushing liquid in and out with a micropipette. The solution was allowed to equilibrate with the surface for 10 to 15 min. The sample was removed from the petri dish and mounted in the fluid cell of the AFM while it remained wet. Before and after collecting the force measurements, we recorded images of the gibbsite platelet, to make sure it had not drifted out of the scanning area. To get data from the various salt species that could be compared, all measurements were performed with the same cantilever. Each time a solution was replaced, the cantilever, the tip holder and the fluid cell were thoroughly flushed with ultrapure deionised water. The tip-sample force was determined from the amplitude and frequency shift vs distance curves using the method presented by Hölscher et al.[2,3].

The measured interaction forces between tip and sample surface were converted to surface charge with Poisson-Boltzmann theory. To optimise accuracy in the fitting process, several points were considered. Rather than using the classical formula for spherical particles, the actual geometry of the tip, derived from SEM images, was used for integrating the stress on the solid surface[4]:

$$F_{el}(D) = \underbrace{P(D)\pi R^2}_{flat\ circular\ area} + \underbrace{2\pi \int_R^\infty P\left(D + \frac{r-R}{\tan\alpha}\right) r dr}_{cone} \qquad \text{(S1)}$$

where $P(D)$ represents the pressure in the gap, $D$ represents the tip-sample distance, $R$, the radius, $\alpha$, the half angle of the tip. A similar expression is obtained for van der Waals part of the interaction for the same geometry

$$F_{vdW}(D) = \underbrace{f(D)\pi R^2}_{flat\ circular\ area} + \underbrace{2\pi \int_R^\infty f\left(D + \frac{r-R}{\tan\alpha}\right) r dr}_{cone} \qquad \text{(S2)}$$

where $f(D) = A_H/(6\pi D^3)$ describes the van der Waals force per unit area for two flat surfaces and $A_H$ is the Hamaker constant. The total tip-sample interaction force is obtained by adding the contributions from the van der Waals and electrostatic interactions, $F_{ts}(D) = F_{el}(D - z_0) + F_{vdW}(D)$. Here we account for a possible shift by $z_0$ of the effective surface separation between the electrostatic interactions originating at the Helmholtz plane, whereas the van der Waals interactions start at the sample surface. To account for the thickness of Stern layers on tip and surface, we used $z_0 = 0.5\text{-}0.6$ nm[5]. More details about theoretical background can be found in ref [4].

Using tabulated values for the Hamaker constants $A_{Gbs} = 1.2\times10^{-20}$ J (gibbsite-water-$SiO_2$)[6,7] and for silica, $A_{SiO2} = 0.65\times10^{-20}$ J ($SiO_2$-water-$SiO_2$)[8], we calculated the AFM tip radius by fitting the model (eq. S2) to the experimental force curves for the highest concentration of divalent salts on silica, for which electrostatic interactions can be neglected. This results in a tip radius of ~56 nm, in agreement with 52±5 nm measured from the SEM images after completion of the AFM experiments.

Tips were initially brought into contact with the surface to blunt them slightly. Force distance curves at high concentrations of divalent cations were conducted several times at the beginning and at the end of each measurement to verify that the tip radius did not vary through the measurement.

Keeping this value fixed, the surface charge as a function of the salt concentration is obtained by fitting the full model curves to the experimental data. In doing so, the symmetric part of the system (silica tip - silica surface) is used to determine the tip charge. Standard expressions are used to calculate the Debye screening length based on the salt concentration. **C**onstant **C**harge (cc) and **C**onstant **P**otential (cp) solutions to the Poisson–Boltzmann (PB) equation are

calculated. The surface charge is determined by fitting the model force curves to the experimental data for tip-sample distances of a few nm, where the cc and the cp solutions overlap.

The heterogeneous nature of the sample allowed us to obtain a calibration of the tip on the silica for every data set, only a few nanometers from the gibbsite particle. This eliminated the need for independent tip calibration.

**DFT:** We used the DMol3 program with the COSMO-RS implicit solvent[9,10,11,12] the PBE[13] functional, the DNP basis set and the dispersion correction by Grimme[14] for the periodic density functional theory (DFT) calculations of the gibbsite {001} basal plane. We used a 1x2 unit cell and the gamma point only for all calculations. We constructed the simulation cell to be three molecular layers thick, perpendicular to the {001} face and the lowest layer was frozen in bulk positions throughout all simulations. All COSMO-RS calculations were performed using the parameterization DMOL3_PBE_C30_1301 in COSMOtherm vC30_1301[15]. All presented geometries were optimized to the medium setting for Dmol3.

All chemical reaction energies included entropy contributions from translational and rotational degrees of freedom for non-slab species, calculated using standard expressions for a gas phase pressure of 1 bar, the same condition used to calculate the solvation energies. Vibrational degrees of freedom were excluded, because of the difficulties involved in calculating these contributions for the slab systems. First, the computation is very expensive, and second, DFT relies on the harmonic approximation for determining the vibrational frequencies. The lowest lying modes can be very anharmonic, and contribute the most to the vibrational entropy. It is therefore very hard to assess the accuracy of this contribution and we have therefore disregarded vibrations in our calculations.

We tested several starting geometries. The water coordination number for adsorbed $Ca^{2+}$ and $Mg^{2+}$ was at least 6 for all calculations and both inner and outer shell adsorption of $Ca^{2+}$ and

$Mg^{2+}$ to the gibbsite basal plane were investigated. All surfaces were neutral. To generate inner shell adsorption complexes, we initialized the calculations by placing two partially dehydrated $Ca^{2+}$ or $Mg^{2+}$ ions per unit cell close to the surface. At the same time, we removed the three more reactive protons from the surface and explicitly added one hydroxyl to keep the unit cell electroneutral. In an alternate test, we left the cations fully hydrated, removed three protons from the hydration water and added one hydroxyl ion per unit cell. Both initial configurations relaxed into the stable zigzag configuration, but the calculated reaction energies clearly favoured the outer shell configuration.

The free hydration energy of the ions in our study was calculated as well using the reference state [1 bar gas / 1 mol solvent], to show that the DMol parameterization in COSMO-RS gives reasonable solvation properties for the ions. The solvation of the divalent cations was determined according to the following reaction:

$$M^{2+} + 6H_2O(aq) \rightarrow M(H_2O)_6^{2+}(aq) \qquad \text{(S3)}$$

Here (aq) means COSMO-RS solvation in water. Without the explicit hydration water, the free energies of solvation of the divalent cations are about 500 kJ/mol too weak. Including waters of hydration clearly improved the simulation, which is why we included them in our adsorption reactions as well, both for the free and the adsorbed ions.

The reaction per unit cell of gibbsite, for outer shell adsorption complexes was:

$$gibbsite(001)\left(H_2O\right)_6(aq) + 2Ca\left(H_2O\right)_6^{2+}(aq) + 4OH^-(aq) \rightarrow gibbsite(001)Ca_2\left(H_2O\right)_6\left(OH\right)_4\left(ads,aq\right) + 12H_2O(aq) \qquad \text{(S4)}$$

To explore the configurations for high salinity solutions, we determined the energy required to exchange one hydroxyl ion in each unit cell for a $Cl^-$ ion, i.e.

$$gibbsite(001)Ca_2\left(H_2O\right)_6\left(OH\right)_4\left(ads,aq\right) + Cl^-(aq) \rightarrow gibbsite(001)Ca_2\left(H_2O\right)_6\left(OH\right)_3Cl\left(ads,aq\right) + OH^-(aq) \qquad \text{(S5).}$$

Equivalent reactions were calculated for $MgCl_2$ as well, Table S1 and S2. The most stable geometries for the Mg structures are shown in Figure S7. The COSMO surface for a bare gibbsite surface is shown in Figure S8 as an example of implicit solvation of a periodic structure.

## II. Tables:

**Table S1:** Reaction energies for outer shell adsorption, which results in the zigzag structure from DFT calculations and the COSMO-RS implicit solvent model. Reactions are written using the stoichiometry for the primitive gibbsite unit cell but the energies are reported per cation. Hydration waters for the gibbsite structures are excluded in the reaction formula for clarity.

| **Reaction** | **ΔG (per $M^{2+}(OH)_2$) (kJ/mol)** | **$[M^{2+}]$ for coverage = 0.5, pH=6 (M)** |
|---|---|---|
| gibbsite(001) + $2Ca(H_2O)_6^{2+}$ + $4OH^- \rightarrow$ gibbsite(001)$Ca_2(OH)_4$(ads)+$12H_2O$ | -118 | $2\cdot10^{-5}$ |
| gibbsite(001) + $2Mg(H_2O)_6^{2+}$ + $4OH^- \rightarrow$ gibbsite(001)$Mg_2(OH)_4$(ads)+$12H_2O$ | -115 | $9\cdot10^{-5}$ |

**Table S2:** Reaction energies for transforming double row structure (low salt concentrations) into single row structure (high conc.) from DFT calculations and the COSMO-RS implicit solvent model. Reactions are written using the stoichiometry per unit cell. Hydration waters for the gibbsite structures are excluded in the reaction formula for clarity.

| **Reaction** | **ΔG (per $Cl^-$) (kJ/mol)** | **$[Cl^-]$ for coverage = 0.5, pH=6 (M)** |
|---|---|---|
| gibbsite(001)$Ca_2(OH)_4$(ads)+ $Cl^- \rightarrow$ gibbsite(001)$Ca_2(OH)_3Cl$(ads) + $OH^-$ | 39 | 0.060 |
| gibbsite(001)$Mg_2(OH)_4$(ads) + $Cl^- \rightarrow$ gibbsite(001)$Mg_2(OH)_3Cl$(ads) + $OH^-$ | 47 | 1.8 |

**Table S3:** Comparison of the experimental free energy of hydration for the ions in this study with our calculations using DFT and COSMO-RS. Energies are in kJ/mol.

| Ion | Experimental[16] | Calculated |
|---|---|---|
| $Mg^{2+}$ | -1830 | -1785 |
| $Ca^{2+}$ | -1505 | -1440 |
| $OH^-$ | -430 | -515 |
| $Cl^-$ | -340 | -369 |

## III. Additional Figures

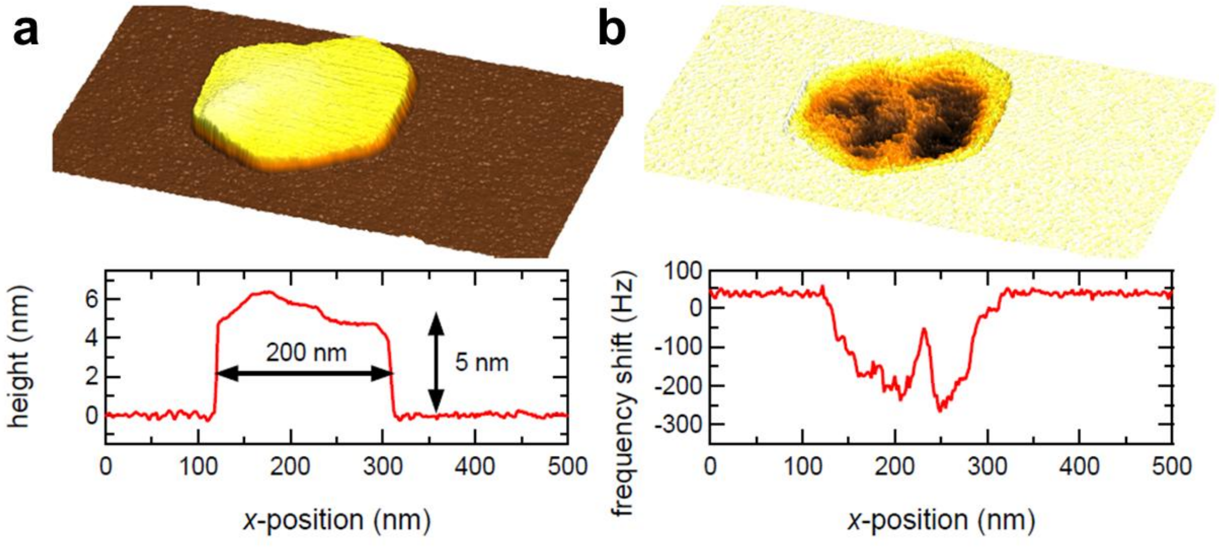


**Figure S1: FM-AFM images of a single gibbsite nanoparticle adsorbed to a silica substrate** **a,** 500×250 nm$^2$ FM-AFM topography image **b,** frequency shift image, each with representative cross section through the gibbsite particle. The nonuniform distribution of frequency shift, revealed by the spike, attributed to a grain boundary running through the crystal. Imaging conditions: 20 mM NaCl solution, CFM Aspire rectangular silicon cantilever, conical tip; $f_0$ = 19.1 kHz, $c_z$ = 4.2 N/m, Q = 9.5.

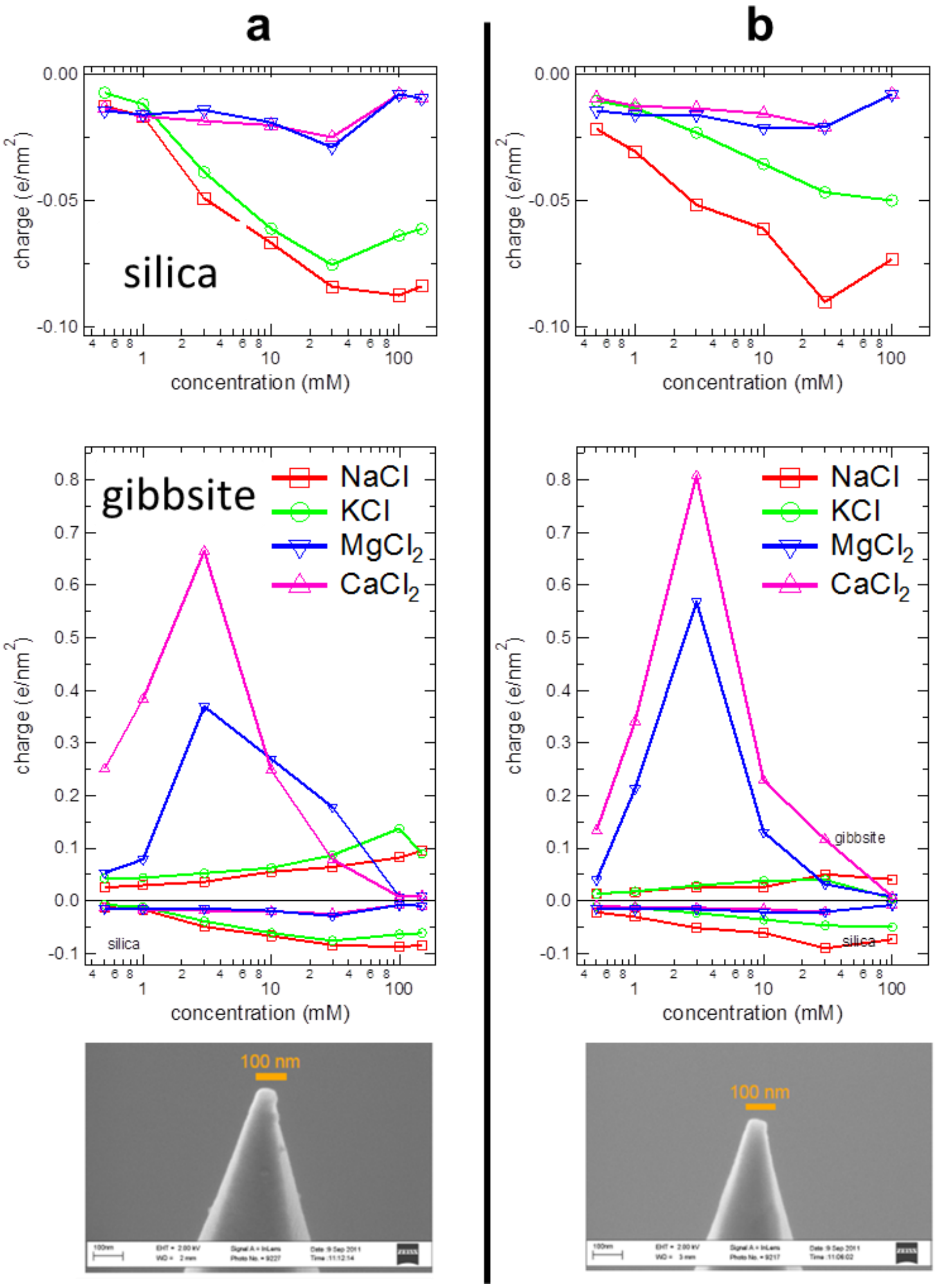


**Figure S2: Effective surface charge vs. solution composition for two other tips on silica and gibbsite.**

**a,** Tip parameters and SEM micrographs for Aspire CFM: $f_0$ = 19.0 kHz, $c_z$ = 4.7 N/m, Q = 11; **b**, and for Aspire CT-130: $f_0$ = 68.4 kHz, $c_z$ = 48.8 N/m, Q = 35. The images show the tips after use.

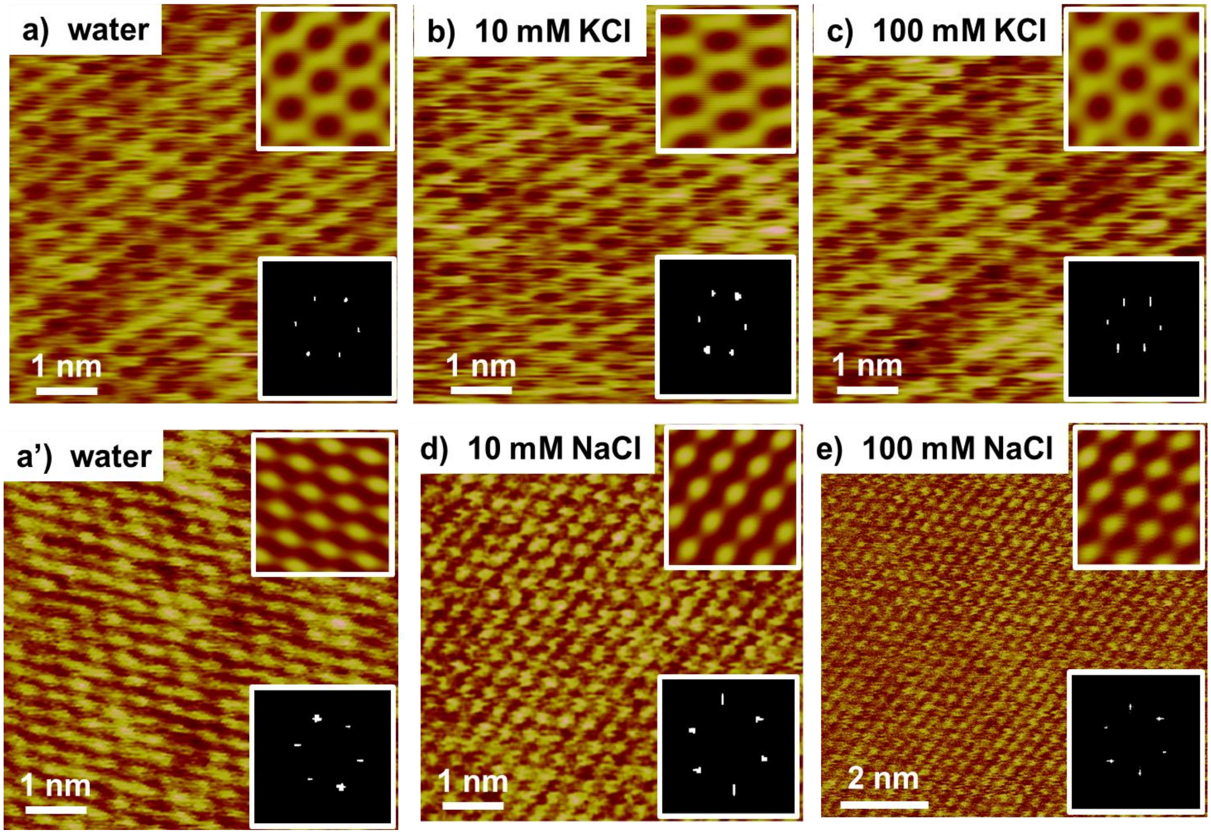


**Figure S3: High resolution, noncontact AM-AFM topographic images of gibbsite during exposure to water and the monovalent cations**

**a,a'**, AFM images of the gibbsite basal plane obtained with super sharp tips at room temperature. The pseudohexagonal structure has 0.5 nm periodicity; **b-e**, Images taken in 10 mM and 100 mM NaCl and KCl solutions show no changes in topography compared with images taken in water. The pattern, with a periodicity of 0.5 nm is the same in all images.

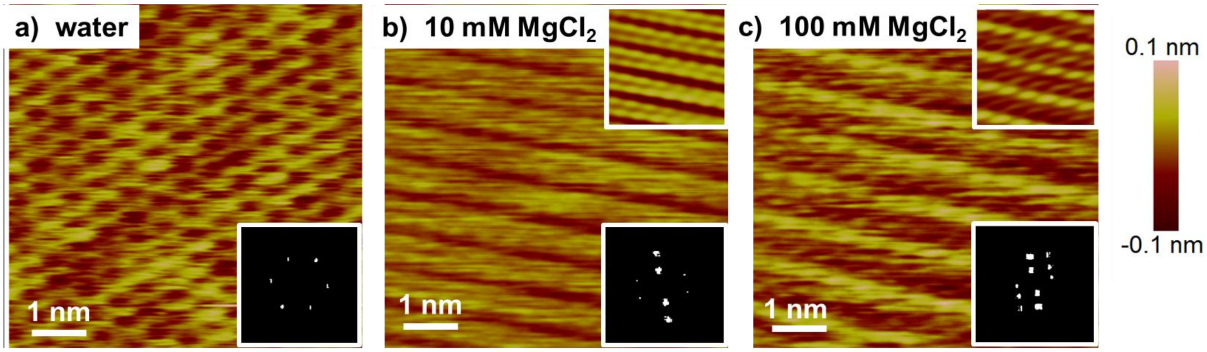


**Figure S4: High resolution, noncontact AM-AFM topographic images of gibbsite, taken in water and $MgCl_2$ solutions.**

The pattern is quite different than that observed in monovalent solutions.

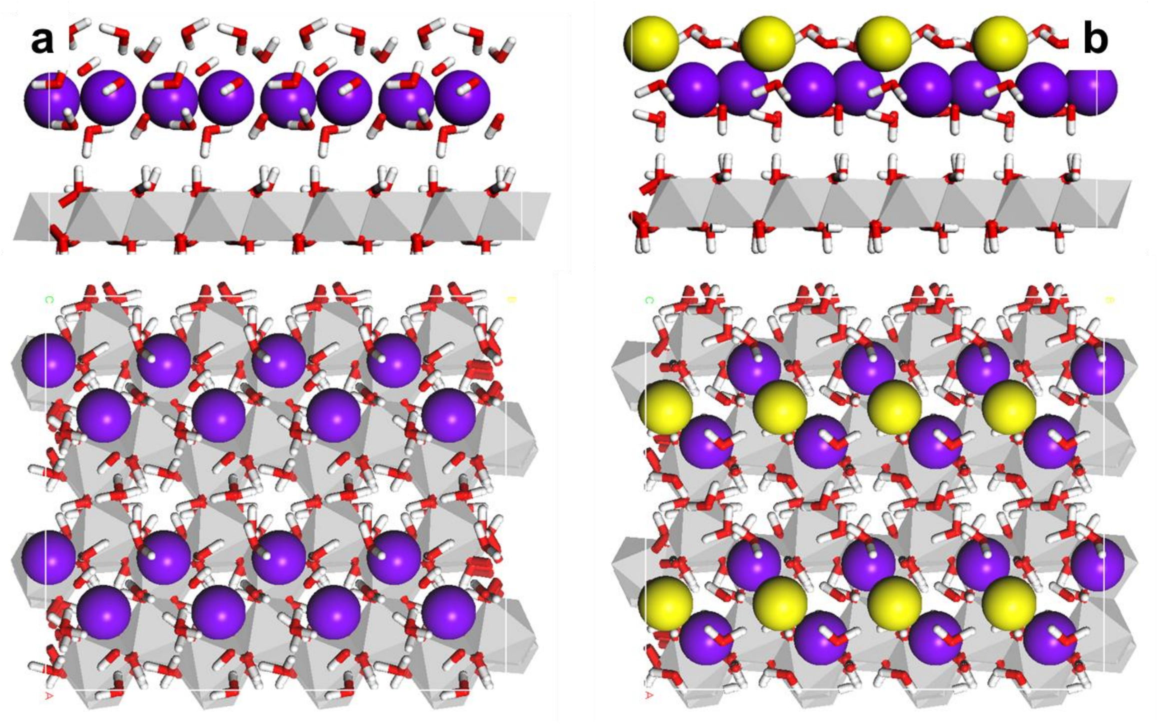


**Figure S5: Structural models produced by DFT simulations**

**a,** $Mg_2(OH)_4$ adsorbed on gibbsite, which produces a zigzag structure and **b**, the same surface but with chloride included, i.e. $Mg_2(OH)_3Cl$, which produces single rows  We have outlined four of the unit cells that we used in the simulations with a white box, which indicates the periodic boundary conditions.

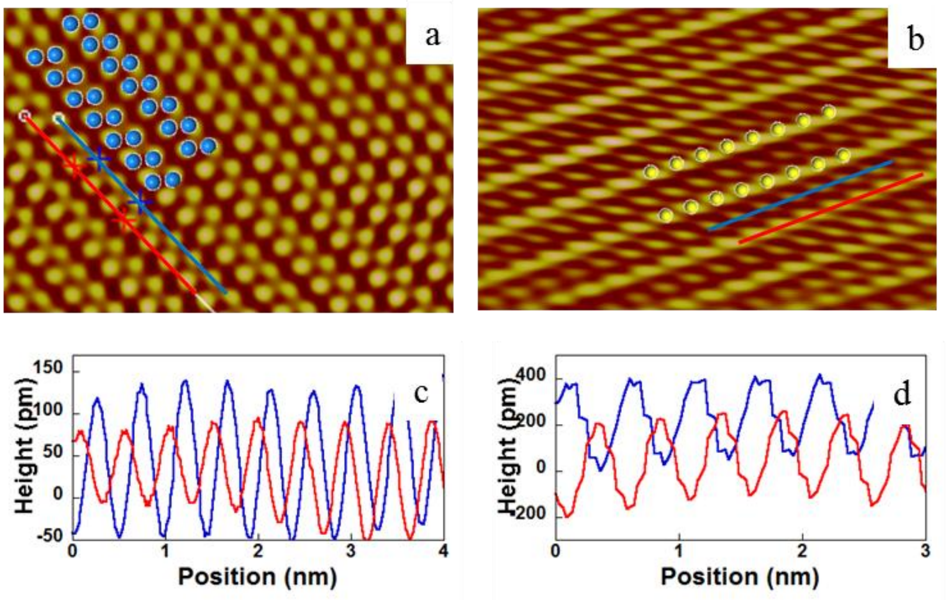


**Figure S6:** AFM topographic images after processing with Fourier transformation taken in **a**, 10 mM and **b,** 100 mM $CaCl_2$ solution (same as Fig 3b and c) superimposed with structural models showing only adsorbed $Ca^{2+}$ (blue) and $Cl^-$ (yelow) ions from Fig 5a and b. **c,** Height profiles are taken as indicated by solid lines in **a,** reveling the distance between the vertical position of the two $Ca^{2+}$ ions which is around <50 pm as predicted by DFT calculations. **d,** Height profiles corresponding to b, showing vertical position of the $Cl^-$ ions which is around 200 pm above the metal cations in agreement with DFT calculations.

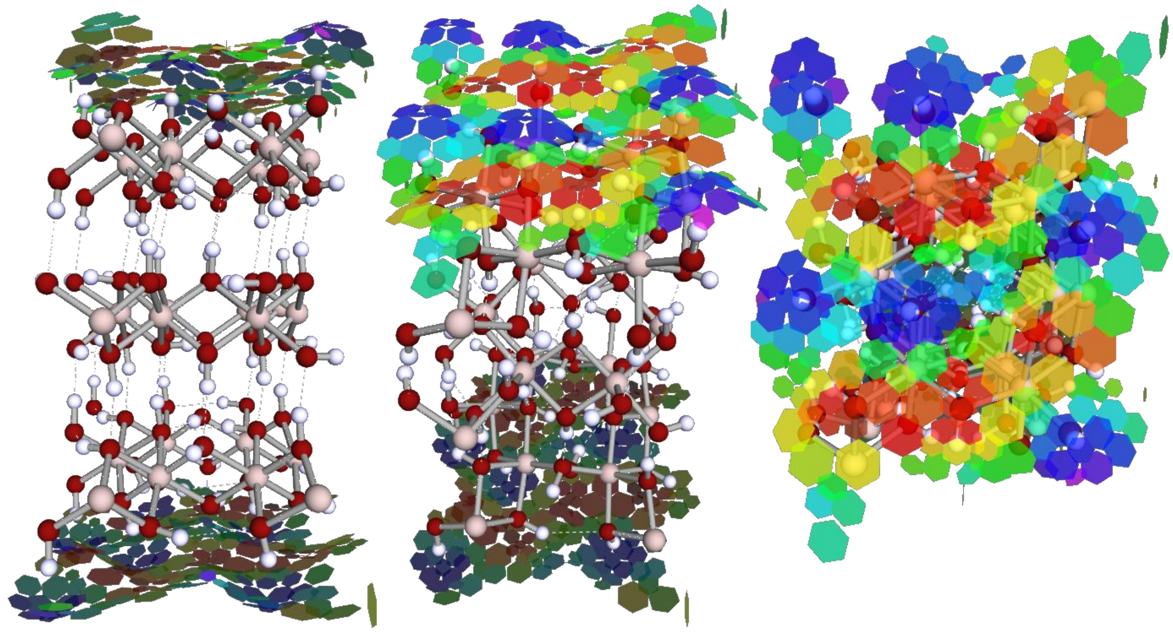

**Figure S7: Molecular structure of a (1x2) unit cell of gibbsite seen from various angles, showing the COSMO surface for the periodic structure.**

Red indicates negative charge and blue, positive; green and yellow are near neutral. Left, side view; right, top view and middle, tilted.

**References:**


1 Wierenga, A. M., Lenstra, T. A. J. & Philipse, A. P. Aqueous dispersions of colloidal gibbsite platelets: synthesis, characterisation and intrinsic viscosity measurements. *Colloids Surf., A* **134**, 359-371 (1998).

2 Ebeling, D. & Hölscher, H. Analysis of the constant-excitation mode in frequency-modulation atomic force microscopy with active Q-Control applied in ambient conditions and liquids. *J. Appl. Phys.* **102** (2007).

3 Ebeling, D., Oesterhelt, F. & Hölscher, H. Dynamic force spectroscopy of single chainlike molecules using the frequency-modulation technique with constant-excitation. *Appl. Phys. Lett.* **95** (2009).

4 Ebeling, D., Van Den Ende, D. & Mugele, F. Electrostatic interaction forces in aqueous salt solutions of variable concentration and valency. *Nanotechnology* **22** (2011).

5 Biesheuvel, P. M. Implications of the charge regulation model for the interaction of hydrophilic surfaces in water. *Langmuir* **17**, 3553-3556 (2001).

6 Gan, Y. & Franks, G. V. Charging behavior of the gibbsite basal (001) surface in NaCl solution investigated by AFM colloidal probe technique. *Langmuir* **22**, 6087-6092 (2006).

7 Butt, H. J., Cappella, B. & Kappl, M. Force measurements with the atomic force microscope: Technique, interpretation and applications. *Surf. Sci. Rep.* **59**, 1-152 (2005).

8 Dishon, M., Zohar, O. & Sivan, U. From repulsion to attraction and back to repulsion: The effect of NaCl, KCl, and CsCl on the force between silica surfaces in aqueous solution. *Langmuir* **25**, 2831-2836 (2009).

9 Klamt, A., Eckert, F. & Arlt, W. COSMO-RS: An alternative to simulation for calculating thermodynamic properties of liquid mixtures. *Annu. Rev. Chem. Biomol. Eng.* **1**, 101-122 (2010).

10 Klamt, A., Jonas, V., Bürger, T. & Lohrenz, J. C. W. Refinement and parametrization of COSMO-RS. *J. Phys. Chem. A* **102**, 5074-5085 (1998).

11 Klamt, A. & Schüürmann, G. COSMO: A new approach to dielectric screening in solvents with explicit expressions for the screening energy and its gradient. *J. Chem. Soc., Perkin Trans. 2*, 799-805 (1993).

12 Delley, B. The conductor-like screening model for polymers and surfaces. *Molecular Simulation* **32**, 117-123 (2006).

13 Perdew, J. P., Burke, K. & Ernzerhof, M. Generalized gradient approximation made simple. *Phys. Rev. Lett.* **77**, 3865-3868 (1996).

14 Grimme, S. Semiempirical GGA-type density functional constructed with a long-range dispersion correction. *J. Comput. Chem.* **27**, 1787-1799 (2006).

15 Eckert, F. & Klamt, A. COSMOtherm Version C3.0, Release 13.01. COSMOlogic GmbH & Co. KG,. (Leverkusen, Germany. , 2013).

16 Marcus, Y. Thermodynamics of solvation of ions. Part 5. - Gibbs free energy of hydration at 298.15 K. *J. Chem. Soc., Faraday Trans.* **87**, 2995-2999 (1991).
